# The Dead or the Living?

**Published:** 1893-10-14

**Authors:** 


### The Hospital

An Institutional Journal of

Science, Medicine, Nursing, and Philanthropy.

*For Hospitals, Asylums, Infirmaries, Dispensaries, Medical Practitioners, Students, and Nurses.*

Vol. XV.—No. 368.] [Oct. 14, 1893.

### THE DEAD OR THE LIVING?

To many medical men the most interesting place in the world is the dead-house. The scientific physician has in his imagination a picture of the internal economy of his patient. At the first consultation he forms a judgment of the condition of the invisible organ or organs whose aberrations he considers to be the starting points of his patient's illness. From week to week, and from month to month, he watches with a curious "internal" eye the processes and structural changes which he believes to be taking place in the diseased organ. As death approaches, in incurable cases, his mental picture is that of a process almost completed, of an organ which has reached such a condition of structural change as to be no longer capable of playing its part in the general economy, and the failure and collapse of which renders life in the organism impossible.

The physician, being after all but a man, has taken a scientific as well as a human and brotherly interest in his patient. He is eagerly curious to know how far the actual condition of the organ whose disease has killed the dead man corresponds with the mental picture he has formed of it during the past few months or years. The dead-house will tell him whether he has been right or wrong. The dead-house proves the physician's competence, as nothing else can prove it.

So profoundly complex is the structure of all the important organs of the human body, and so mysterious and interesting are many of the processes which lead to disease, disintegration, and death, that not a few of the ablest minds in the medical profession voluntarily turn away from the possibilities of honour and emolument which lie before them in every-day practice, and devote themselves through all the months and years to the study of the diseased, dead, and decaying organs of the body. These men are not to be pitied. They have a task of infinite complexity. Success on a considerable scale is their all-sufficient reward. And it is well that it should be so; for the great world can never be made to understand the nature of their work sufficiently to take a lively interest in it; and, therefore, their only reward must be their own satisfaction in their work, and the approval of the strictly limited number of their professional brethren who sympathetically and intelligently follow their steadfast labours. For our own part, we value the workers in the dead-house, and their work, as the traveller in a strange land values those trusted guides who save him from innumerable dangers, and conduct him with safety and honour over at least some part of the half-lighted pathway which lies between him and the end of his journey.

But the question is now being asked whether the dead-house has still in its possession any secrets which have not been fully revealed? Some not incompetent persons are inclined to say that it has not. The careful dissection, the trained naked eye inspection, the microscope with its low powers and its high powers, the various stains with their excellent capacities of differentiation, have all been used with such interminable repetition and on so world-wide a scale that it is affirmed that with our present methods the dead-house has nothing more to reveal. If morbid anatomy has more secrets hid in her profound depths, new methods must be devised if the world is not to remain ignorant of them.

"The living," is the cry which the younger pathologists are raising on all hands. "The living," is the cry which practising medical men re-echo from the consulting-rooms of large towns and the fever-stricken and phthisis-ridden abodes of our villages and farms. The dead-house tells us where diseases end; we want to know where they begin. The difficulties of this problem are immense. Many, even among practising medical men, have no faintest conception of their nature and extent. How, then, can the uninstructed public know or sympathise? And yet it is the public which ought to know of these difficulties, and which ought to sympathise with us in our efforts to elucidate them. For how shall we discover the beginnings of disease, except by infinitely repeated observations upon those living beings in whom the diseases have their origin, and at the earliest stages of disease origination? If we desire to know all about any particular thing, we must study that particular thing; that and nothing else.

What we want then is to study living human forms; to study them when ill, and to study them when well; above all, to study them in the earliest stages of disease. But how are we to study them? Do we not study them already? Is not every effort made to obtain at the very outset the fullest and most minute knowledge of every disease process which is associated with mankind? We are not prepared to say that every possible effort is made. The methods of to-day are not the methods of a century ago; still less are they the methods of the period of the Norman Conquest. If we have methods infinitely superior to those even of the most competent physicians of ancient times, why may not our successors, why may not we ourselves, devise newer and more efficient methods than those which our teachers have handed down to us?

It is a fact of deep significance that a man of such a keen and powerful intellect as John Hunter devoted thirty of the best years of his life to one study, the study of the blood. Was he working aimlessly in the dark all those years? Or had he a great and all-illuminating purpose? Without doubt Hunter saw clearly the object which he had in view. "Let me but unravel all the secrets of the blood," he seemed to say, "and everything else will be clear before me." We want a full and true science of the living blood. Its composition, of course, we know, and many other things about it; but a full and complete science of the blood, the living blood, we certainly do not possess. Who among us examines the blood of our patients as an ordinary part of diagnosis and treatment? We all make a point of systematically examining the urine; but who, we repeat, makes a point of systematically examining the blood? Besides, the general physician has no scientific standard whereby to work, no intimate knowledge of living blood under every possible physiological and pathological condition. Much as we know of the blood, the physiology of it as a whole from birth to death, and the pathology of it as a whole from the beginning to the ending of every known disease, constitute as yet two comparatively unknown worlds.

The blood, it can hardly be doubted, holds the secrets of the beginnings of disease. But it is the living human blood. Whatever experiments we may make on animals, the knowledge derived from such experiments can never be the same as knowledge derived from the actual chemical, microscopical, experimental, and detailed study of living human blood, drawn at every period of life, and at every stage of every disease, by competent physiologists and physicians in every country. Who will furnish the practising medical profession with a full and working science of the blood, in youth and age, in men and women, in health and disease?

Medicine is for the living: it is not for the dead. Whatever physiology or morbid anatomy may be, medicine is a practical art. The curious scientist may be happy in the gratification of his curiosity; but medicine in the large sense owes it to itself and to the world to mitigate human ills, to lessen, and, where possible, to abolish suffering, and to make life happy, successful, and long. Nothing short of this, both as an aim and as a performance, can place medicine on a level with the other great professions and sciences which elevate and ameliorate the lot of man. Give us science, science, and evermore science; but above all things give us that practical knowledge which will enable us to justify our existence and our honours and rewards to a much-tired, much-toiling, and much-suffering world.

